# USP10 alleviates sepsis-induced acute kidney injury by regulating Sirt6-mediated Nrf2/ARE signaling pathway

**DOI:** 10.1186/s12950-021-00291-7

**Published:** 2021-08-19

**Authors:** Fei Gao, Mingjiang Qian, Guoyue Liu, Wanping Ao, Dahua Dai, Cunzhi Yin

**Affiliations:** 1 Department of Critical Care Medicine, Affiliated Hospital of Zunyi Medical University, 563003 Zunyi City, Guizhou Province China; 2 Department of Critical Care Medicine, The Second Affiliated Hospital of Zunyi Medical University, Intersection of Xinlong Avenue and Xinpu Avenue, Xinpu New District, Honghuagang District, 563000 Zunyi City, Guizhou Province China

**Keywords:** USP10, SIRT6, CLP, NRF2, ARE, AKI

## Abstract

**Background:**

Severe sepsis, a major health problem worldwide, has become one of the leading causes of death in ICU patients. Further study on the pathogenesis and treatment of acute kidney injury (AKI) is of great significance to reduce high mortality rate of sepsis. In this study, the mechanism by which ubiquitin specific peptidase 10 (USP10) reduces sepsis-induced AKI was investigated. Ligation and perforation of cecum (CLP) was employed to establish C57BL/6 mouse models of sepsis. Hematoxylin-eosin (H&E) staining was performed to detect renal injury. The concentrations of serum creatinine (Cr), urea nitrogen (BUN) and cystatin C (Cys C) were determined using a QuantiChrom™ Urea Assay kit. RT-qPCR and western blot were conducted to assess the USP10 expression level. DHE staining was used to detect reactive oxygen species (ROS) levels. H_2_O_2_, MDA and SOD levels were assessed using corresponding colorimetric kits. Western blot was used to examine the expression levels of Bcl-2, Bax, cleaved caspase-3, Sirt6, Nrf2 and HO-1. MTT assay was used to determine cell viability, whereas TUNEL staining and flow cytometry were used to assess cell apoptosis.

**Results:**

In this study, we found that USP10 was decreased in CLP-induced mouse renal tissues. We identified that USP10 alleviated renal dysfunction induced by CLP. Moreover, USP10 was found to reduce oxidative stress, and abated LPS-induced renal tubular epithelial cell injury and apoptosis. Finally, we discovered that USP10 promoted activation of the NRF2/HO-1 pathway through SIRT6 and attenuated LPS-induced renal tubular epithelial cell injury.

**Conclusions:**

This study found that USP10 activates the NRF2/ARE signaling through SIRT6. USP10 alleviates sepsis-induced renal dysfunction and reduces renal tubular epithelial cell apoptosis and oxidative stress.

## Background

Severe sepsis and septic shock are major health problems worldwide and have become one of the leading causes of death in ICU patients [1]. Acute kidney injury (AKI) is the most common complication among sepsis due to multiple organ failure [2, 3]. Endothelial dysfunction and microcirculation disturbance caused by endothelial cell apoptosis are the hallmarks of sepsis [4]. However, there is currently no effective therapy for AKI, and the mechanism that leads to AKI remains unclear [5]. Thus, further study on the pathogenesis and treatment of AKI is of great significance to reduce high mortality rate of sepsis.

Ubiquitin specific peptidase 10 (USP10) is a member of the deubiquitinase family that removes ubiquitin from proteins tagged for lysosomal degradation, thereby preventing their removal and degradation. Previous studies have shown that USP10 acts as an anti-stress factor against a variety of environmental stresses, including oxidative stress, viral infection and heat shock [6]. For example, USP10 expression is decreased and upregulated in hepatic steatosis models to inhibit hepatic steatosis, insulin resistance, and inflammation [7]. Low expression of USP10 induced by cerebral ischemia/reperfusion is associated with brain injury, and increased USP10 expression has a protective effect against cerebral ischemia injury by inhibiting inflammation and apoptosis through blocking TAK1 signal transduction [8]. USP10 is expressed in both sepsis and acute respiratory syndrome patients [9]. However, the role of USP10 in sepsis-induced organ damage is unclear, AKI in particular, which requires further investigation.

Studies have shown that the protective effect of USP10 against organ injury is related to SIRT6. USP10 interacts with SIRT6 to inhibit its ubiquitination and degradation, thereby preventing fatty liver and cardiac hypertrophy[7]. Notably, SIRT6 overexpression alleviates AKI caused by sepsis [10]. Furthermore, SIRT6 is known to reduce apoptosis and oxidative stress in vascular endothelial cells by promoting the activation of NRF2/ARE signaling pathway [11]. In this study, we examined the expression of USP10 in kidney tissues of sepsis-induced AKI mouse models, and explored the function of USP10 in sepsis-induced renal dysfunction and renal tubular epithelial cells. In addition, the role of USP10 in sepsis-induced oxidative stress in renal tissues via SIRT6-mediated NRF2 /ARE signaling pathway was investigated.

## Results

### USP10 is decreased in CLP-induced mouse renal tissues

**Fig. 1:**
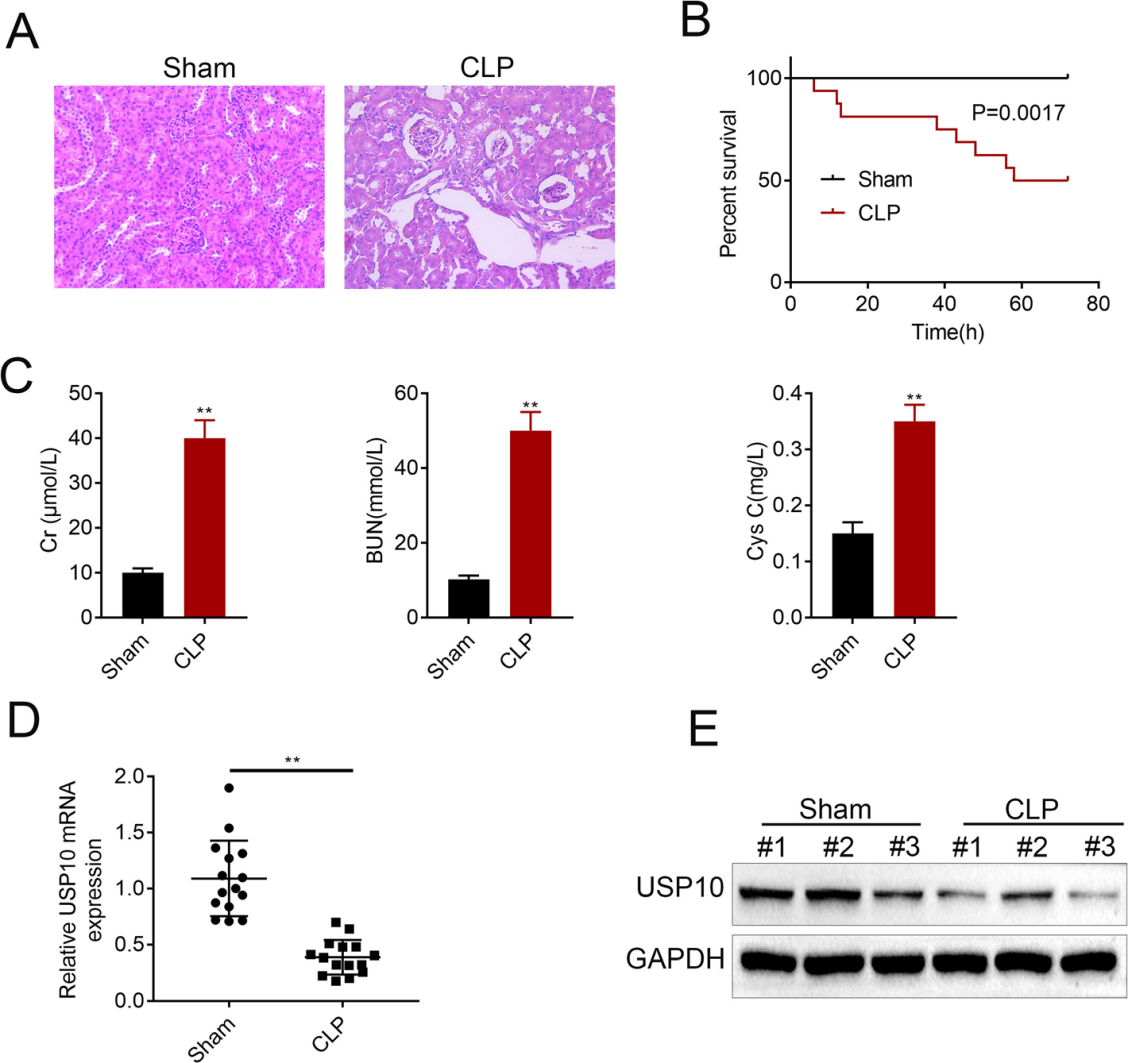
USP10 is decreased in CLP-induced mouse renal tissues. (**A**) H&E images show the cell morphology and inflammatory changes in renal tissues of the CLP and sham groups (n = 15). (**B**) Graph shows 72-hour survival rates. (**C**) The concentrations of Cr, BUN and Cys C were examined at 24 h after CLP. The mRNA (**D**) and protein (**E**) expression levels of USP10 were analyzed in the CLP and sham groups, **p < 0.01. GAPDH was used as an internal control

Firstly, we established C57BL/6 mouse models of sepsis using CLP. H&E staining was performed to detect renal injury and the 72-hour survival rate was calculated. The morphology and organization of renal tissues in the sham group were normal, whereas marked pathological changes, including severe vacuolar degeneration in renal tubular epithelial cells, detachment of renal tubular epithelial cells and infiltration of inflammatory cells (Fig. 1 A) were observed in the renal tissues of CLP mice. The survival rate declined to 50 % at 72 h after CLP compared to 100 % survival rate in the sham group (Fig. 1B). Then, we examined the concentrations of serum creatinine (Cr), urea nitrogen (BUN) and cystatin C (Cys C) in the sham and CLP groups. We found significant increase in the concentrations of Cr, BUN and Cys C in the CLP group compared to the sham group (Fig. 1 C). Furthermore, both of the mRNA (Fig. 1D) and protein (Fig. 1E) levels of USP10 were decreased in the CLP group compared to the sham group. These results reveal that USP10 is downregulated in CLP-induced AKI mouse models of sepsis.

### USP10 alleviates renal dysfunction induced by CLP

**Fig. 2:**
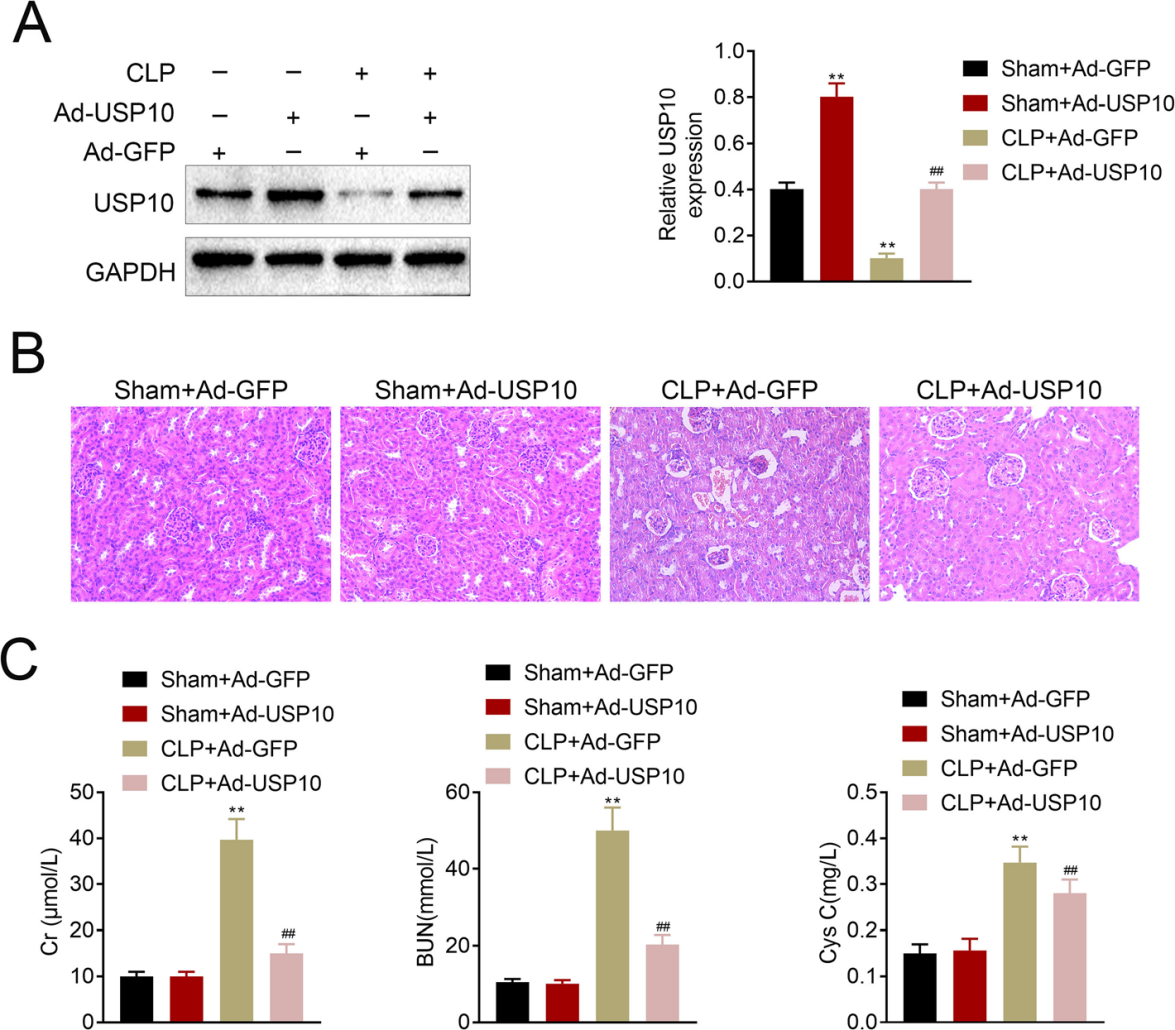
USP10 alleviates renal dysfunction induced by CLP. (**A**) Western blot images show the transfection efficiency of USP10, **p < 0.01. GAPDH was used as an internal control. (**B**) H&E images show the cell morphology and inflammatory changes in renal tissues of the Sham + Ad-GFP, Sham + Ad-USP10, CLP + Ad-GFP and CLP + Ad-USP10 groups (*n* = 15). (**C**) The concentrations of Cr, BUN and Cys C were examined at 24 h after CLP in the Sham + Ad-GFP, Sham + Ad-USP10, CLP + Ad-GFP and CLP + Ad-USP10 groups, **p < 0.01

In order to validate the function of USP10 in CLP-induced AKI, we transfected the human renal tubular epithelial cells and divided into four experimental groups named Sham + Ad-GFP, Sham + Ad-USP10, CLP + Ad-GFP and CLP + Ad-USP10. Results from the western blots confirmed the transfection efficiency of USP10, which showed significant upregulation of USP10 levels in the Sham + Ad-USP10 and CLP + Ad-USP10 groups compared to the Sham + Ad-GFP and CLP + Ad-GFP groups, respectively (Fig. 2 A). H&E staining results demonstrated milder tubular epithelial cell exfoliation and focal tubular epithelial vacuolar degeneration with fewer inflammatory cells in the CLP + Ad-USP10 group compared with the CLP + Ad-GFP group (Fig. 2B). Moreover, the concentrations of Cr, BUN and Cys C were decreased in the CLP + Ad-USP10 group compared to the CLP + Ad-GFP group (Fig. 2 C). Based on these findings, we inferred that USP10 alleviates renal dysfunction induced by CLP.

### USP10 attenuates CLP-induced oxidative stress in renal tissues

**Fig. 3:**
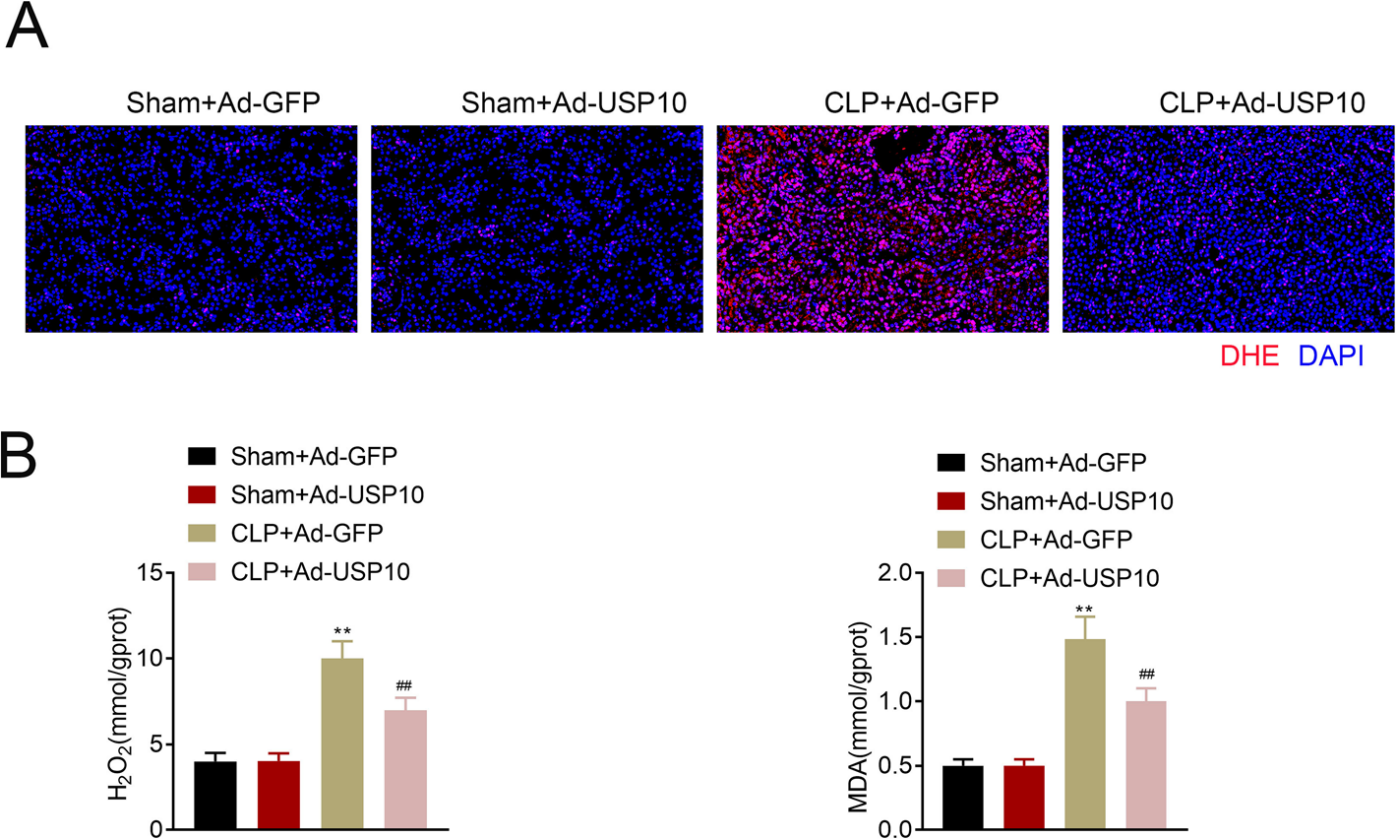
USP10 attenuates CLP-induced oxidative stress in renal tissues. (**A**) Representative DHE staining images of the Sham + Ad-GFP, Sham + Ad-USP10, CLP + Ad-GFP and CLP + Ad-USP10 groups demonstrating ROS levels, bar = 20 μm. (**B**) Comparison of H_2_O_2_ and MDA levels in renal tissues (*n* = 8) of the Sham + Ad-GFP, Sham + Ad-USP10, CLP + Ad-GFP and CLP + Ad-USP10 groups using the colorimetric kits. **p < 0.01

ROS levels were detected by superoxide anion fluorescent probe DHE staining. The images showed no discernible difference in DHE distribution between the Sham + Ad-GFP and Sham + Ad-USP10 groups. However, DHE distribution was markedly decreased in the CLP + Ad-USP10 group compared to the CLP + Ad-GFP group (Fig. 3 A). The renal levels of H_2_O_2_ and MDA in four groups were determined using the colorimetric kits, which showed an increase in H_2_O_2_ and MDA levels in the CLP + Ad-GFP and CLP + Ad-USP10 groups compared to that of Sham + Ad-GFP and Sham + Ad-USP10 groups. Furthermore, the levels of H_2_O_2_ and MDA were reduced in the CLP + Ad-USP10 group compared to the CLP + Ad-GFP group (Fig. 3B). Altogether, these results illustrate that USP10 attenuates CLP-induced oxidative stress in renal tissues.

### USP10 reduces CLP-induced apoptosis in renal tissues

**Fig. 4:**
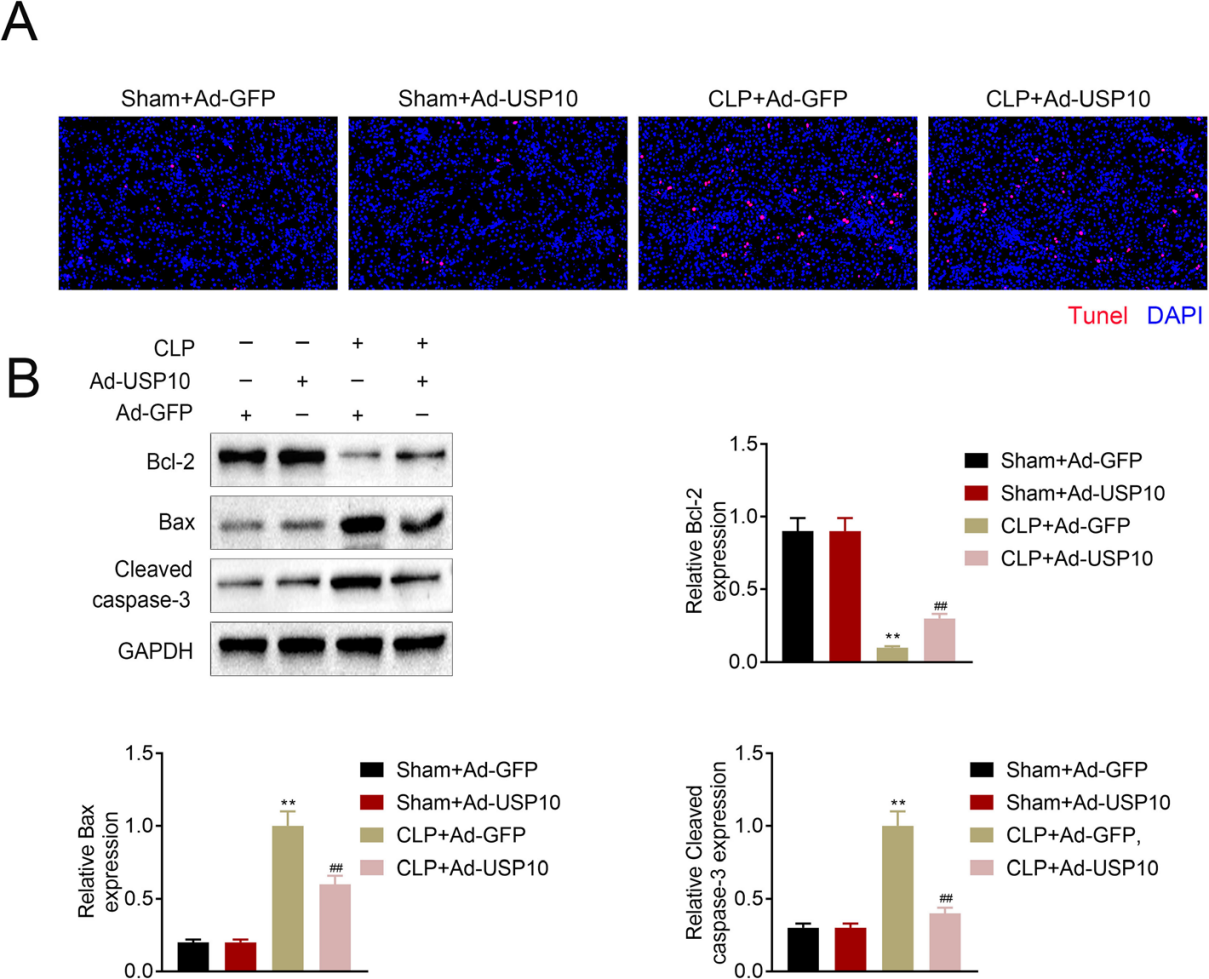
USP10 reduces CLP-induced apoptosis in renal tissues. (**A**) Representative TUNEL staining images of renal tissues of the Sham + Ad-GFP, Sham + Ad-USP10, CLP + Ad-GFP and CLP + Ad-USP10 groups (*n* = 8) indicating the apoptotic rate, bar = 20 μm. (**B**) Western blot images demonstrating Bcl-2, Bax, and cleaved caspase-3 expression levels in renal tissues of the Sham + Ad-GFP, Sham + Ad-USP10, CLP + Ad-GFP and CLP + Ad-USP10 groups, **p < 0.01. GAPDH was used as an internal control

Furthermore, we employed TUNEL assay and western blot to determine cell apoptosis in CLP-induced renal tissues. Images showed an increased apoptotic rate in the renal tissues after CLP induction, and was reduced by USP10 overexpression (Fig. 4 A). Moreover, western blot analysis demonstrated decreased Bcl-2 protein level, while Bax and Cleaved caspase-3 were increased in the CLP + Ad-GFP and CLP + Ad-USP10 groups compared to the Sham group. In the CLP group, overexpression of USP10 boosted the level of Bcl-2 and suppressed Bax and cleaved caspase-3 (Fig. 4B). These outcomes indicate that USP10 inhibits CLP-induced apoptosis in renal tissues.

### USP10 abates LPS-induced renal tubular epithelial cell injury

**Fig. 5:**
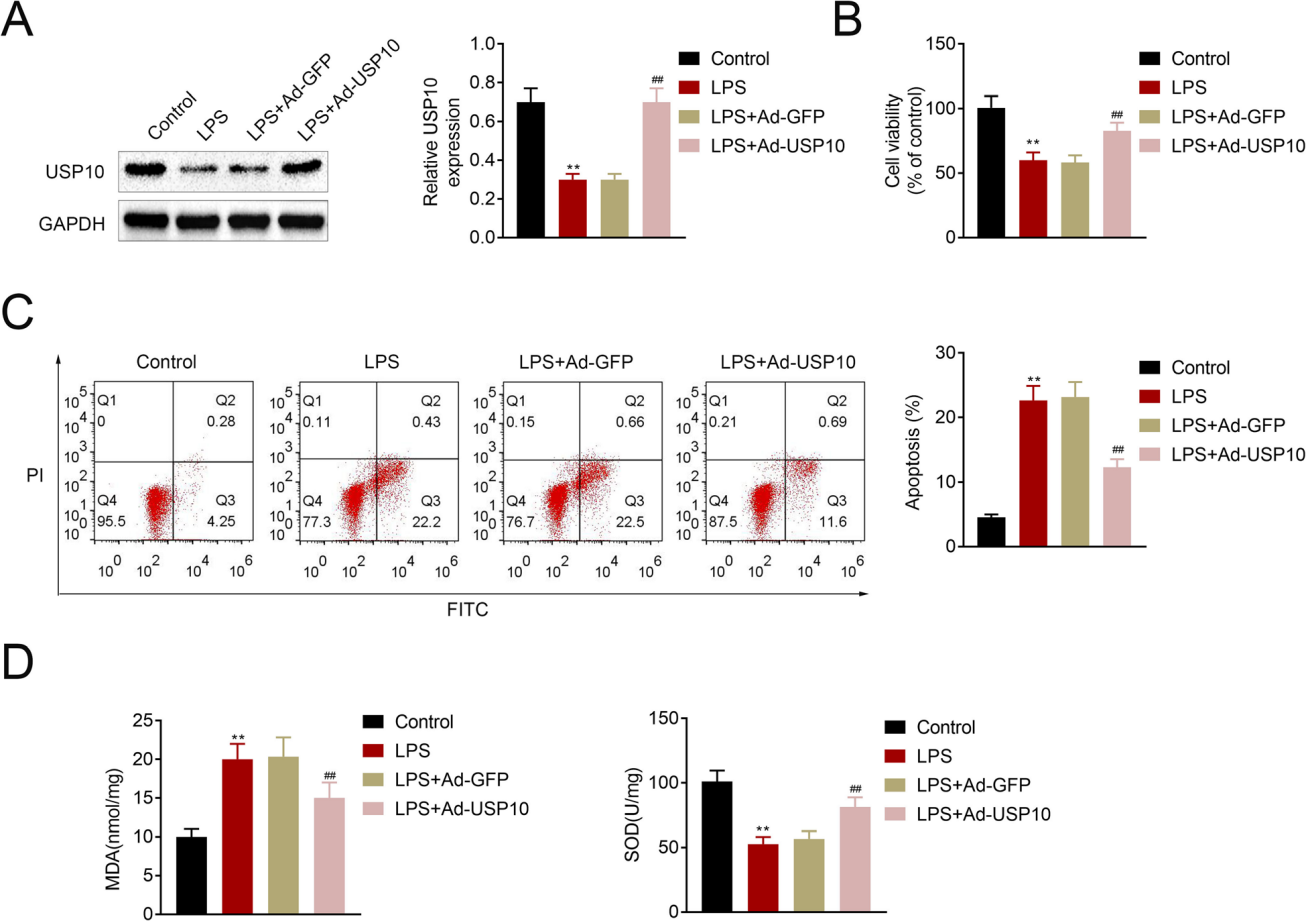
USP10 attenuates LPS-induced renal tubular epithelial cell injury. (**A**) Western blot images showing USP10 protein levels in the control, LPS, LPS + Ad-GFP and LPS + Ad-USP10 treated human renal tubular epithelial cell line, HK-2, **p < 0.01. GAPDH was used as an internal control. (**B**) MTT assay shows cell viability in control, LPS, LPS + Ad-GFP and LPS + Ad-USP10 HK-2 cells, **p < 0.01. (**C**) Flow cytometry charts show cell apoptosis in control, LPS, LPS + Ad-GFP and LPS + Ad-USP10 HK-2 cells, **p < 0.01. (**D**) Comparison of MDA and SOD levels in four HK-2 treatment groups using the colorimetric kits, **p < 0.01

Human renal tubular epithelial cell line, HK-2, was used in this study. Western blot showed a marked decrease in USP10 in the LPS group compared to control mice, and USP10 was significantly upregulated in the LPS + Ad-USP10 group compared to the LPS + Ad-GFP group (Fig. 5 A). MTT assay and flow cytometry revealed that LPS induction led to decreased cell viability and increased cell apoptosis, which was reversed by USP10 overexpression (Fig. 5B-C). Furthermore. MDA and SOD levels were determined using MDA and SOD assay kits, respectively, and the results showed that LPS induction led to increased MDA and reduced SOD, which was reversed when USP10 was overexpressed (Fig. 5D). These findings suggest that USP10 abates LPS-induced renal tubular epithelial cell injury.

### USP10 promotes activation of the NRF2/HO-1 pathway through SIRT6 and attenuates LPS-induced renal tubular epithelial cell injury

**Fig. 6:**
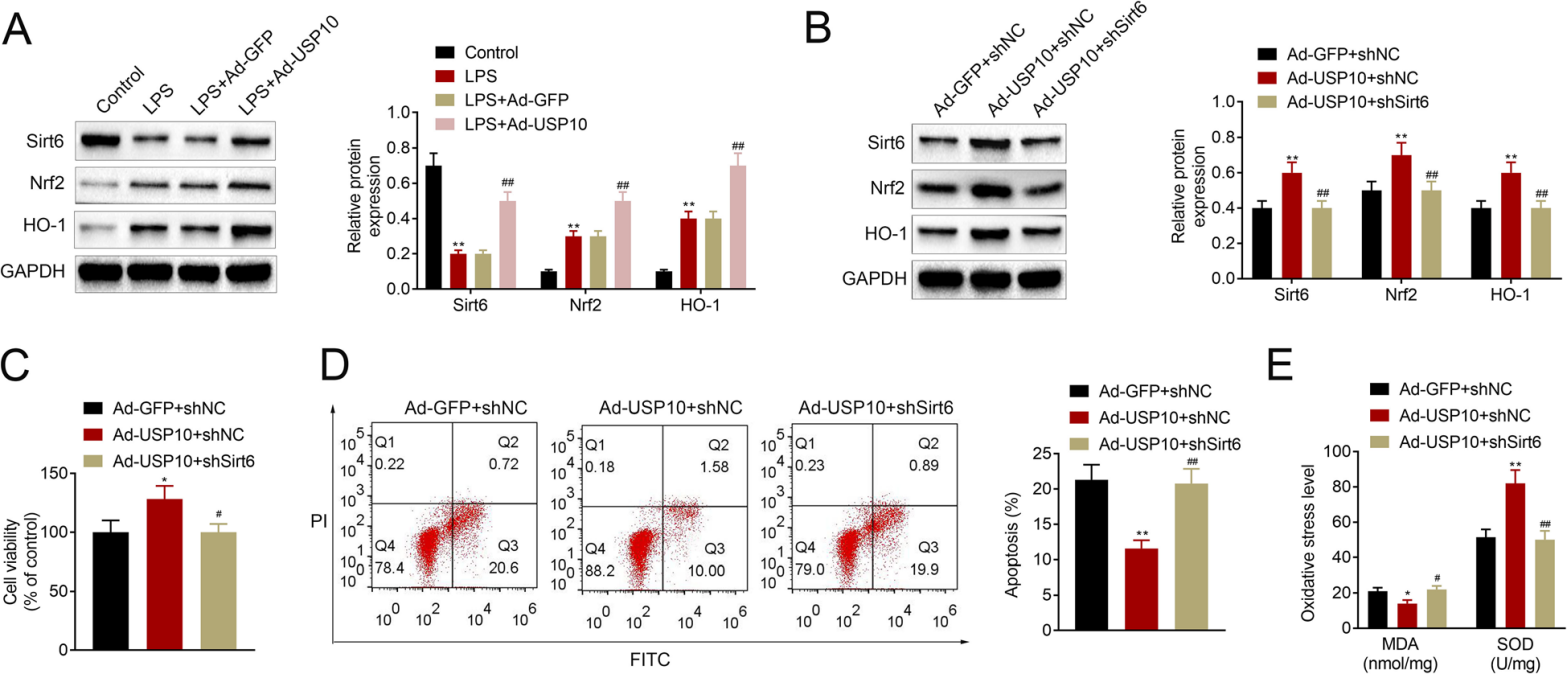
USP10 promotes activation of the NRF2/HO-1 pathway through SIRT6 and attenuates LPS-induced renal tubular epithelial cell injury. (**A**-**B**) Western blot images show the expression levels of Sirt6, Nrf2 and HO-1 in control, LPS, LPS + Ad-GFP, LPS + Ad-Sirt6, Ad-GFP + shNC, Ad-USP10 + shNC and Ad-USP10 + shSirt6 HK-2 cells, **p < 0.01. GAPDH was used as an internal control. (**C**) MTT assay demonstrating cell viability in Ad-GFP + shNC, Ad-USP10 + shNC and Ad-USP10 + shSirt6 HK-2 cells, *p < 0.05. (**D**) Flow cytometry charts showing cell apoptosis in Ad-GFP + shNC, Ad-USP10 + shNC and Ad-USP10 + shSirt6 HK-2 cells, **p < 0.01. (**E**) Comparison of MDA and SOD levels in Ad-GFP + shNC, Ad-USP10 + shNC and Ad-USP10 + shSirt6 HK-2 cells using the colorimetric kits, *p < 0.05, **p < 0.01

Previous studies have shown that the protective effect of USP10 against organ injury is mediated through SIRT6 [7], and SIRT6 is known to attenuates apoptosis and oxidative stress through activating the NRF2/ARE signaling pathway [11]. This prompted us to further interrogate the relationship between USP10 and Sirt6 and the Nrf2/HO-1 signaling pathway. Western blot assay showed that LPS resulted in decreased Sirt6 and increased Nrf2 and HO-1 levels, whereas USP10 overexpression led to an increased expression of Sirt6, Nrf2 and HO-1 in HK-2 cells induced by LPS (Fig. 6 A). Furthermore, our results demonstrated that USP10 overexpression increased Sirt6, Nrf2 and HO-1 levels, but were reduced following Sirt6 knockdown (Fig. 6B). MTT assay and flow cytometry revealed increased cell viability and reduced cell apoptosis, respectively, in the Ad-USP10 + shNC group, and this was reversed by Sirt6 knockdown in the Ad-USP10 + shSirt6 group (Fig. 6 C-D). MDA level was also decreased and SOD was increased in the Ad-USP10 + shNC group, while in the Ad-USP10 + shSirt6 group, increased MDA level and reduced SOD level were observed (Fig. 6E). Collectively, these results suggest that USP10 promotes activation of the NRF2/HO-1 pathway through SIRT6 and attenuates LPS-induced renal tubular epithelial cell injury.

## Discussion

Sepsis-induced acute kidney injury (AKI) is a common complication in critically ill patients. The prevention of AKI is difficult because most patients have already developed AKI when they seek medical attention [12]. To date, no effective therapy is available to relieve acute kidney injury or expedite recovery [13]. Studies have identified various biological substances that can reduce AKI. For example, Quercetin was found to abate AKI through suppressing ferroptosis [14]; Uncoupling protein 1 reduces AKI through inhibiting mitochondrial reactive oxygen species generation [15]; Inhibition of pannexin-1 attenuates sepsis-induced AKI by reducing cell apoptosis and NLRP3 inflammasome activation [16, 17]; and aquaporin 1 blocks AKI through PI3K-mediated macrophage M2 polarization [18]. Our study reports for the first time that USP10 alleviates sepsis-induced renal dysfunction, and reduces renal tubular epithelial cell apoptosis and oxidative stress.

USP10 is closely related to many biological processes. USP10 is a critical regulator of KLF4 stability and suppresses lung tumorigenesis [19]. USP10 promotes hepatocellular cancer metastasis by stabilizing Smad4 protein [20]. Downregulated USP10 alone or combination of USP10/p14ARF are robust indicators of poor prognosis in ovarian cancer patients [21]. USP10 suppresses lung cancer cell invasion and growth via upregulating PTEN [22]. Moreover, USP10 has been reported to exert its function through multiple signaling pathways. For example, USP10 regulates Notch signaling pathway in the endothelium [23]; USP10 protects against cerebral ischemia injury by attenuating inflammation and apoptosis through inhibiting the TAK1 signaling pathway [8]; Interaction of USP10 and G3BP2 blocks p53 signaling and results in poor prognosis in prostate cancer [24]; Wu-5, which is an inhibitor of USP10, enhances crenolanib-induced FLT3-ITD-positive AML cell death by suppressing FLT3 and AMPK signaling pathways [25]. Furthermore, previous study has demonstrated that deubiquitinating enzyme is related to fibrosis [26]. In agreement with this, in this study, we found that overexpression of USP10 inhibited fibrosis in LPS-induced mouse models. Our study revealed that USP10 attenuated sepsis-induced oxidative stress in kidney tissues and activated the NRF2/ARE signaling pathway. Thus, our findings suggest that USP10 may be a potential therapeutic target in sepsis-induced AKI. However, it remains unknown whether DUB inhibitors that target USP10 could make the recipients of other clinical conditions more susceptible to sepsis and this warrants further investigation.

Sirt6 is one of the members of sirtuin family. Recently, complex signaling networks regulated by Sirt6 and are common to many clinical organ injuries have been identified [27]. Sirt6 deficiency exacerbates proteinuria and podocyte injury by targeting Notch signaling pathway [28]. Sirt6 overexpression abates cisplatin-induced AKI through suppressing ERK1/2 signaling [29]. Sir6 protects retinal ganglion cells from oxidative stress-induced damage through activating the Nrf2/ARE signaling pathway and inhibits Bach1 in retinal degenerative diseases [30]. Sir6 promotes cerebral ischemia reperfusion injury by binding with miR-370 and regulates the Nrf2/ARE signaling pathway [31]. In the present study, we found that Sirt6-mediated Nrf2/ARE signaling alleviates sepsis-induced AKI via USP10.

## Conclusions

In conclusion, our findings show that USP10 expression is decreased in kidney tissues of sepsis-induced AKI mouse models. USP10 overexpression alleviates sepsis-induced renal dysfunction and reduces damage in renal tubular epithelial cells. Moreover, USP10 attenuates sepsis-induced oxidative stress in renal tissues by increasing SIRT6 stability and activating the NRF2 /ARE signaling pathway. Altogether, these findings illustrate that USP10 alleviates sepsis-induced AKI by regulating Sirt6-mediated Nrf2/ARE signaling pathway, proposing a novel target for the clinical treatment of AKI.

## Methods

### Cell culture and transfection

HK-2 cells were grown in Dulbecco’s Modified Eagle Medium (DMEM; Biological Industries, Kibbutz Beit Haemek, Israel) supplemented with 10 % fetal bovine serum (FBS; Biological Industries, Kibbutz Beit Haemek, Israel), 1 % penicillin/streptomycin (Gibco), and 0.025 µg/ml amphotericin B (Sigma-Aldrich, St. Louis, MO, USA). Cells were cultured in a humidified 5 % CO_2_ incubator at 37 °C. Adenovirus pDC316 vector (Genecreate, China) was used to overexpress USP10. Adenovirus was harvested through lysing 293 A cells.

### Sepsis-induced CLP mouse model

All animal experiments in this study were conducted in accordance with the Guide for the Care and Use of Laboratory Animals [32] and approved by the Ethics Committee of Affiliated Hospital of Zunyi Medical University. C57BL/6 mice (male, 8–10 weeks old) were subjected to CLP to induce sepsis. All mice were anesthetized with isoflurane. A 2-cm midline laparotomy was made to expose the caecum under aseptic conditions. Then the caecum was ligated by a 4 − 0 silk suture and punctured twice using a 20-gauge needle. After surgery, all mice received 1 ml warm saline via intraperitoneal injection and were placed in individual cages.

### MTT assay

MTT was used to assess cell viability. Briefly, cells (2.5 × 10^3^ cells/ml) were seeded into 96-well plates in triplicates and treated in different conditions as indicated in each experiment. Following treatment, a final concentration of 0.5 mg/mL of MTT solution (Beyotime) was added to each well, and cells were incubated in MTT solution for another 4 h at 37 °C. Then, culture medium was discarded and 100 µL of dimethyl sulfoxide (DMSO, Sigma) was added to dissolve the formazan crystals formed from the reactions. The optical density (OD) value of each sample was detected at 490 nm through a microplate reader (BioTek, Winooski, VT, USA).

### Cell apoptosis

Annexin V/FITC and PI apoptosis detection kit (Sigma-Aldrich, St. Louis, Carlsbad, CA, USA) was used to determine cell apoptosis. Flow cytometry was performed using a BD Accuri™ C6 (CA, USA). Briefly, cells were digested, washed, and resuspended in Annexin V incubation solution. Then cells were kept in dark for at least 30 min, 37 °C and quantified through flow cytometry.

### RNA extraction and quantitative real-time polymerase chain reaction (qPCR)

Total RNA was extracted from collected cells by adding the Trizol reagent (Invitrogen, CA, USA). The purity and concentration of extracted total RNA were determined by a Nano Drop 1000 spectrophotometer (Thermo Fisher Scientific, Grand Island, USA). The ChamQTM SYBR® qPCR Master Mix (Vazyme, Nanjing, China) was used to amplify the USP10 cDNAs on a QuantStudio 6 Flex Real-Time PCR System (Life Technologies, Carlsbad, California) according to manufacturer’s instructions. The expression values of target gene were normalized to the U6 expression. PCR primers were designed and synthesized by Tsingke Technology (Beijing, China). Relative expression of USP10 in each experimental group was analyzed using the 2^−△△Ct^ method [33, 34]. Primer sequences are listed in Table 1. All experiments were performed in triplicates.

**Table 1:** Primers for USP10 and reference genes

Gene	Primer	Sequence(5’→3’)
USP10	Forward	ATTGAGTTTGGTGTCGATGAAGT
	Reverse	GGAGCCATAGCTTGCTTCTTTAG
U6	Forward	TCCTCCACGACAACCAAAACC
	Reverse	TCTTTTCCCAAAATCCCAGACTC
β-actin	Forward	GTGACGTTGACATCCGTAAAGA
	Reverse	GCCGGACTCATCGTACTCC

### Histological examination

Paraffin-embedded kidney tissues were cut into 4-µm sections. For H&E staining, sections were deparaffinized using xylene and stained with hematoxylin for 5 min at room temperature. After washing, sections were incubated with Eosin for 2 min. For DHE assay, samples were cut into 5-µm sections, then DHE (10 mol/L) was added. Slides were incubated in a light-protected humidified chamber at 37 °C, for 30 min. Ethidium fluorescence was examined under a fluorescence microscope. For TUNEL assay, sections were dewaxed and dehydrated. After inactivation of endogenous peroxidases, sections were incubated with TUNEL reaction solution for 1 h at 37 ℃. Finally, 2 % DAB developing solution was added and sections were visualized under a fluorescence microscope.

### Western blot

Cells were washed three times in pre-cooled PBS buffer, and total protein was extracted using RIPA buffer (Beyotime, Shanghai, China). Protein concentrations were quantified using a BCA protein assay kit (CoWin Biotechnology). Equal amounts of total proteins were loaded and electrophoresed by SDS-PAGE. Then, proteins were transferred onto polyvinylidene difluoride membranes (PVDF; Millipore) and blocked with 5 % non-fat milk at room temperature for 1 h. Proteins were detected by incubating with specific primary antibodies: rabbit anti-USP10 antibody (ab109219, 1:5000; Abcam, Cambridge, MA, USA), rabbit anti-Bcl-2 antibody (ab182858, 1:2000; Abcam, Cambridge, MA, USA), rabbit anti-Bax antibody (ab32053, 1:5000; Abcam, Cambridge, MA, USA), rabbit anti-cleaved caspase-3 antibody (ab32042, 1:500; Abcam, Cambridge, MA, USA), rabbit anti-Sirt6 antibody (ab191385, 1:2000; Abcam, Cambridge, MA, USA), rabbit anti-Nrf2 antibody (ab62352, 1:500; Abcam, Cambridge, MA, USA), rabbit anti-HO-1 antibody (ab189491, 1:2000; Abcam, Cambridge, MA, USA), and rabbit anti-GAPDH antibody (ab8245, 1:5000; Abcam, Cambridge, MA, USA) overnight at 4 °C. Then, membranes were incubated with HRP-conjugated goat anti-rabbit immunoglobulin G secondary antibody (ab205718, 1:1500; Abcam). Protein bands were visualized using the ECL chemiluminescence reagent (Beyotime) and quantified by gray scale analysis using the ImageJ software (National Institutes of Health). GAPDH was used as a housekeeping reference.

### Colorimetric assays

Cr, BUN and Cys C levels were determined using a QuantiChrom™ Urea Assay kit (DIUR-500, Hayward, CA) according to manufacturer’s instructions.

### Oxidative stress analysis

Blood samples were collected in lithium-heparin tubes and were centrifuged at 1,500 × g for 10  minutes. Plasma was separated and stored at -80 °C until use. Index of oxidative injury was determined by the levels of malondialdehyde (MDA) using an MDA assay kit (A003-1-2, Nanjing Jiancheng Bioengineering Institute, Nanjing), whereas the antioxidant capacity was indicated by the levels of superoxide dismutase (SOD) using a SOD assay kit (A001-3-2, Nanjing Jiancheng Bioengineering Institute, Nanjing). The readings were measured by spectrophotometry.

### Statistical analysis

All data are presented as mean ± standard deviation from three independent experiments. Student’s t-test was used to compare between two groups. *p* values of < 0.05 were considered as statistically significant.

## Data Availability

All data generated or analyzed during this study are included in this published article.

## Abbreviations

AKI: Acute kidney injury
USP10: Ubiquitin specific peptidase 10
CLP: Ligation and perforation of cecum
H&E: Hematoxylin-eosin
Cr: Creatinine
BUN: Urea nitrogen
Cys C: Cystatin C
ROS: Reactive oxygen species
